# The extracellular vesicles secreted by lung cancer cells in radiation therapy promote endothelial cell angiogenesis by transferring miR-23a

**DOI:** 10.7717/peerj.3627

**Published:** 2017-08-25

**Authors:** Yongfa Zheng, Liang Liu, Cong Chen, Pingpo Ming, Qin Huang, Changhu Li, Dedong Cao, Ximing Xu, Wei Ge

**Affiliations:** 1 Renmin Hospital, Wuhan University, Wuhan, Hubei, China; 2 Shanghai Cancer Center, Fudan University, Shanghai, Shanghai, China; 3 Taihe Hospital, Hubei University of Medicine, Shiyan, Hubei, China

**Keywords:** EV, Radiation therapy, Lung cancer cells, Angiogenesis, miR-23a

## Abstract

Angiogenesis is an important factor contributing to the radioresistance of lung cancer. However, the associated mechanisms underlying radiotherapy-induced pro-angiogenesis are unclear. Here, we demonstrated that Extracellular vesicles (EVs) derived from cultured cells in vitro enhanced HUVEC proliferation and migration, and the enhancement effect became more obvious when HUVECs were treated with EV derived from A549 or H1299, two lung cancer cell lines. Additionally, the pro-angiogenesis effect induced by EV could be strengthened when the lung cancer cells were exposed to X-ray irradiation. Furthermore, we verified that the downregulation of PTEN plays a vital role in this process. By evaluating the changes in the levels of microRNAs(miRNAs) targeting PTEN in EV, we found that miR-23a was significantly upregulated and mediated a decrease in PTEN. A luciferase reporter gene transfer experiment demonstrated that PTEN was the direct target of miR-23a, and the kinetics of PTEN expression were opposite to those of miR-23a. Our results show that the miR-23a/PTEN pathway plays an important role in EV-induced angiogenesis. These findings implicate the miR-23a/PTEN axis as a novel therapeutic target for lung cancer radiotherapy.

## Introduction

Lung cancer has one of the highest morbidity and mortality rates of all cancers and has a poor prognosis (Torre et al., 2012). Similar to the treatment modalities for most solid tumors, the therapeutic approaches to lung cancer cells include radiotherapy, alone or in combination with surgery or chemotherapy (Polanski et al., 2016; Hellmann et al., 2016). Recently, molecular targeted therapies for lung cancer have demonstrated efficacy, but the 5-year survival rate of lung cancer patients continues to be dismal (Chaffer & Weinberg, 2011); therefore, it is urgent to elucidate the mechanisms of radiation resistance, and identify new therapeutic targets that will increase the cytotoxicity of radiotherapy.

Previous studies have shown that in radiation-resistant cells, pathways that can increase cell survival, such as the protein kinase B (PKB/AKT) and extracellular regulated protein kinases (ERK) pathways, are activated (Li et al., 2016a, 6. Pak et al., 2004). In addition, radiation may induce abnormal expression of some miRNAs that may function as oncogenes (Verma & Lautenschlaeger, 2016). These changes may decrease the levels of lethal factors or tumor suppressors, and promote the proliferation of cancer cells. It has been reported that lung cancer-related chemoradiation resistance can lead to tumor recurrence and metastasis (Ochiai et al., 2016). Therefore, identifying the mechanisms contributing to radiotherapy-induced metastasis will reduce the mortality rate and increase the disease-free survival of patients diagnosed with lung cancer.

Angiogenesis plays crucial roles in various physiological processes, including wound healing and tissue repair. Previous studies have shown that tumor metastasis also depends on angiogenesis (Folkman, 1995). Providing the neoplasm with nutrition, the newly formed vessels lead to the rapid proliferation and epithelial-mesenchymal transition (EMT) of cancer cells, and ultimately tumor metastasis occurs (Li et al., 2016b; Kim et al., 2016). It has been demonstrated that, by increasing the formation of blood vessels (Choi et al., 2016), radiotherapy could decrease the sensitivity of cancer cells to the treatment (Bow et al., 2014; Donneys et al., 2015). Anti-angiogenesis treatment could strengthen the cytotoxicity of chemotherapy or radiotherapy to lung cancer (Yoshimura et al., 2013; Fang & Declerck, 2013). Therefore, anti-angiogenesis plays an important role in oncotherapy. However, the detailed mechanism that can promote tumor angiogenesis has not been analyzed thoroughly. It has been reported that multiple factors, such as phosphatase and tensin homolog on chromosome ten (PTEN), vascular endothelial growth factor (VEGF), transforming growth factor beta (TGF-β), and its transcription factor hypoxia-inducible factor-1(HIF-1), contribute to angiogenesis (Sakurai & Kudo, 2011; Chen et al., 2016; Krishnan et al., 2015; Martinez-Lara et al., 2016). PTEN is a phosphatase with a central function in counteracting phosphatidylinositol AKT and ERK pathways (Putz et al., 2012), playing a key role in angiogenesis by inhibiting endothelial cell proliferation (Kessler et al., 2015). Recently, EVs secreted by tumor cells have also been reported to contribute to angiogenesis (Qi et al., 2016; Liang et al., 2016).

EVs are small, membraned vesicles (30∼100 nm) that originate from the inward budding of the endosomal membrane (Liang et al., 2016). EVs can carry into target cells complex biological information, including mRNAs, microRNAs (miRNAs) and soluble proteins (Raposo & Stoorvogel, 2013; Valadi et al., 2007), which can modify the biological function of the target cells. Depending on the type of the target cells and the functions of the transferred molecules, the biological effects on the target cells can vary. In angiogenesis, human umbilical vein endothelial cells (HUVECs) take up the EVs secreted by tumor cells so that they induce proliferation and migration of HUVECs. Several studies have demonstrated that the underlying mechanism of these effects most likely occurs through miRNAs (Xu et al., 2015). For example, transferred miR-125 could promote the formation of vessels by decreasing the level of DLL4 (Liang et al., 2016). Additionally, EVs can also affect angiogenesis by transforming reactive oxygen species (ROS) to target cells (Alcayaga-Miranda et al., 2016).

Here, we suggest that miR-23a, transferred by EVs, can promote the proliferation and migration of HUVECs and accelerate angiogenesis in radiotherapy through inhibiting the expression of PTEN. This may be one of the causes of radiation resistance in lung cancer and provide a new target for increasing the sensitivity of radiotherapy.

## Materials and Methods

### Cells

The lung cancer cell lines A549 and NCI-H1299 cells, HEK293 and endothelial cells (HUVECs) were purchased from American Type Culture Collection (ATCC). A549, HEK293 and endothelial cells were cultured in Dulbecco’s Modified Eagles Medium (DMEM) medium (Gibco, American) and NCI-H1299 cells were cultured in Roswell Park Memorial Institute (RPMI1640) medium (Gibco, American) supplemented with 10% fetal bovine serum (FBS). Media used for all cell lines were supplemented with antibiotics (penicillin and streptomycin). All cultures were maintained at 37 °C in a 5% CO_2_ incubator. HUVECs were exposed to HEK293, A549 and NCI-H1299 EVs (0.02 mg/ml culture medium).

### EVs isolation

EVs were isolated from cell culture medium by differential ultracentrifugation according to previous works (Zhang et al., 2010). Briefly, cell culture supernatants were collected when cells have grown up to about 70% confluence. Then, the collected supernatants were centrifuged at 200× g for 10 min to remove residual cells followed by centrifuging again at 2,000× g for 20 min to remove other debris. Finally, the supernatants were centrifuged at 100,000× g for 70 min to pellet the EVs. The EVs pellet was washed twice and resuspended in 200 µl phosphate buffered saline (PBS). All operations were performed at 4 °C . The EVs from radiation-exposed lung cancer cells were collected after exposure to 4 Gy X-ray for 72 h.

To study the uptake of lung cancer cell-released EVs by HUVECs, EVs were labeled with the fluorescent probe PKH-26 using ac labeling kit (Sigma) as described previously (Medina & Ghahary, 2010). Briefly, 100  mg protein equivalents of EV pellets were resuspended in 1 ml PBS prior to mixed with 1 ml of PHK26 probe diluted in diluent C (1:1 v/v). The mixture was incubated for 5 min and diluted with 4.5 ml PBS. To pellet the PKH-26-labeled EVs, ultracentrifugation at 100,000× g for 70 min were performed. The labeled EVs pellet was then washed twice with PBS to ensure removal of any free dye. And finally, the labeled EVs pellet was re-suspended in 100 µl PBS and used for further uptake studies.

### Electron microscopy

EVs were analyzed by negative-staining transmission electron microscopy (TEM) as reported previously (Booth et al., 2006). Firstly, purified EVs pellet (2.5 ml) was adsorbed onto ForEVar/carbon-coated copper mesh grids, rinsed in filtered PBS, and then stained with freshly prepared 2.0% phosphotungstic acid in aqueous suspension. Images for samples were acquired using a JEM-1230 TEM (JEOL, Japan) equipped with a LaB6 cathode, operated at an acceleration voltage of 80 kV.

### Western blot

Antibodies used in our research included: CD63 (Abcam, ab59479), total AKT (Abcam, ab8805), phosphorylation AKT (Abcam, ab38449), JAK(Abcam, ab108596), YAP(Abcam, ab52771), β-catenin(Abcam, ab32572), p53(Abcam, ab26), p21 (Abcam, ab109520), p-ERK(Abcam, ab214362), ERK(Abcam, ab54230), GAPDH (Santa Cruz, sc-81545). Cells and EVs were lysed in non-denaturing cell lysis buffer (consists of 20 mM Tris HCL pH 8, 137 mM NaCl, 10% glycerol, 1% NP-40, 2 mM EDTA, protease and phosphatase inhibitors), followed by sonication and incubated on ice for 30 min before centrifugation at 14,000× g for 30 min at 4 °C. The equal amounts of protein for each sample were re-suspended in sample buffer and resolved by sodium dodecylsulfate polyacrylamide gel electrophoresis (SDS-PAGE), then transferred to polyvinylidene fluoride (PVDF) membranes. The membranes were immunoblotted with the indicated antibodies against CD63 (1: 500), total AKT (1: 500), p-AKT (1: 500);, GAPDH(1: 1,000), then followed by secondary antibodies which has been conjugated to horseradish peroxidase (HRP). Bands were further scanned and quantitated using the Quantity One (Bio-Rad) imaging system. To test protein concentration in lysates, a BCA protein assay kit (Beyotime) was carried out. Loading proteins from cells and EVs were both 50 µg.

### Cell viability assay

To test the cell viability, cells of different groups were seeded in 96-well plates and given relative treatments for indicated times, then a Cell Counting 8 kit (CCK-8; Dojindo) was carried out. After incubated for 1.5 h in room temperature, the OD value at 450 nm was detected according to the manufacturer’s instruction.

### Transwell assays

Transwell assays were used to measure cellular migration as described previously (Huttenlocher, Ginsberg & Horwitz, 1996). Briefly, HUVECs were seeded in the upper chambers without the matrigel membrane covered in 24-well plates. After incubation at 37 °C, 5% CO_2_ for 24 h, the lower chamber was observed using an inverted microscope. The incubation was terminated when the cells passed into the lower chamber, then taken out the upper chambers and cleaned them with a cotton swab. The lower chambers were washed with PBS, and fixed them with 4% paraformaldehyde, followed by 0.1% crystal violet staining, Finally, chambers were washed three times with distilled water, and then photographed.

### Wound healing assay

Cells were seeded in six-well plates to measure two-dimensional movement as in previous job (Morales et al., 1995). A wound was created with a sterile pipette tip in the middle of a confluent plate. Next, the medium was replaced with DMEM medium without FBS. Photographs were captured immediately or after 36 h using an Olympus BX51 microscope, and the wound distance in 0 h was calculated as a basic width. Wound closure (%) was determined by the width migrated after 36 h ratio to the basic width.

### Detection of miRNAs

Detection of miRNAs were referenced in a previous work (Sayed et al., 2007). Briefly, miRNAs in cells and EVs were isolated using a miRcute miRNA Isolation Kit (TIANGEN Biotechnology), and the levels of miRNAs were tested using a miRNA qRT-PCR Detection System (GeneCopoeia, AOMD-Q020). Expression of miRNAs was normalized to RNU6-2/snRNA U6-2 (RNA, U6 small nuclear 2). The relative expression levels of the miRNAs were determined with the 2^−ΔΔCT^ method.

### Luciferase reporter assay

Luciferase reporter assay was performed to verify miRNA regulate ability as previous work (Wu et al., 2008). Briefly, HUVEC cells were seeded in 48-well plates and grown to 70∼80% confluence. Then, the cells were cotransfected with pMIR-PTEN and the monitor plasmid pRL-TK (Promega, E2241). After treated 36 h, the cells were lysed, and the Firefly and Renilla luciferase activities were measured using a Dual-Luciferase Reporter System (Promega, E1910) according to the manufacturer’s instructions. The transfection experiments and relative luciferase reporter assays were performed at least three times in triplicate. Finally data are represented as the fold change after normalizing the luciferase activity of the tested sample to that of the corresponding control sample.

### Statistical analysis

All experiments were carried out in triplicate and repeated at least twice. The data are expressed as mean + SD and the significance of the results was determined using Student’s *t*-test and Anova. Differences were considered significant when *P* < 0.05.

## Results

### Isolation and characterization of EVs

**Figure 1:**
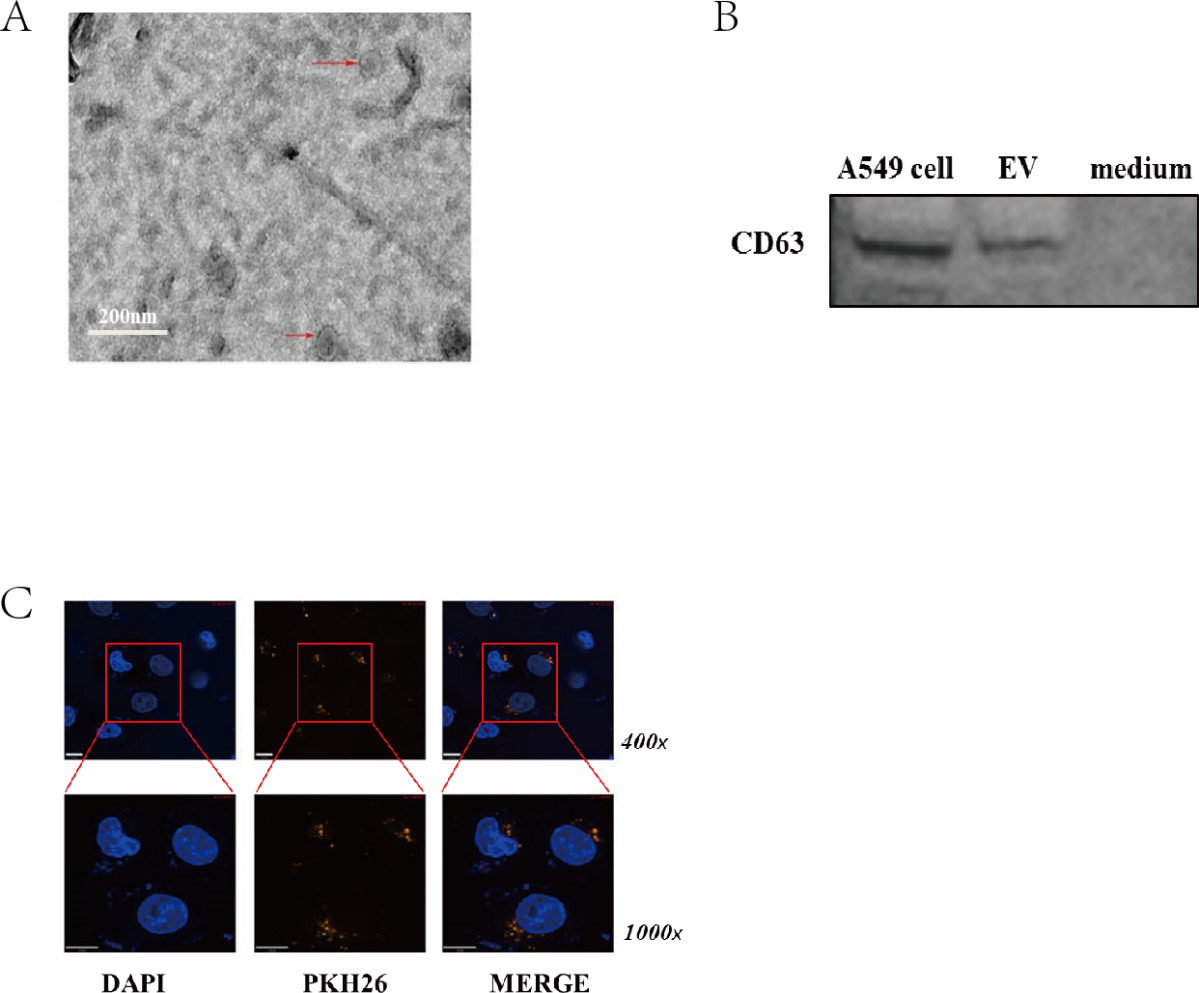
Characterization of EV. (A) Electron micrograph of EVs isolated from A549-conditioned medium. Arrowheads indicate EVs. Scale bar: 200 nm. (B) CD63 expression was detected in EVs. Western blotting was performed with the lysates of EVs, A549 cells as positive control, and medium as negative control. (C) HUVECs were incubated with PKH-26-labeled EVs (PKH-26 is shown in yellow), and uptake of EVs was observed in HUVECs. The nuclei of endothelial cells were stained with DAPI (blue). Arrowheads indicate PKH-26-labeled EVs within endothelial cells. Scale bars: 50 µm.

To ensure that the isolated pellets were genuine EVs, the collected pellet from A549 cell culture medium was captured under a transmission electron microscope (TEM) to identify their shape and analyzed marker of EVs by western blotting. Representative TEM images of EVs obtained from the supernatant of A549 cells are shown in Fig. 1A. A homogeneous population of round vesicles (40–100 nm in diameter) was observed. CD63, a member of the tetraspanin family, is an evolutionarily conserved protein in EVs and a widely used biomarker for testing EVs. Western blotting was performed to further confirm that the collected pellets were EVs by detecting the presence of CD63 in the samples derived from A549 cells, A549 cells as positive control and cell culture medium as negative (Fig. 1B). To further investigate the transportability of EVs, we labeled EVs with the fluorescent dye PKH-26 and then incubated them with HUVECs *in vitro*. Next, fluorescence microscopy was taken to confirm the uptake. After incubated for 6 h, more than 90% of HUVECs were PKH-26 signal positive, which means that EVs could be taken up and transferred to recipient cells (Fig. 1C). Taken together, these data suggest that EVs secreted by lung cancer cells can be successfully isolated and can be transferred to HUVECs efficiently.

### The EVs isolated from irradiation-exposed lung cancer cells increase the proliferation of endothelial cells

**Figure 2:**
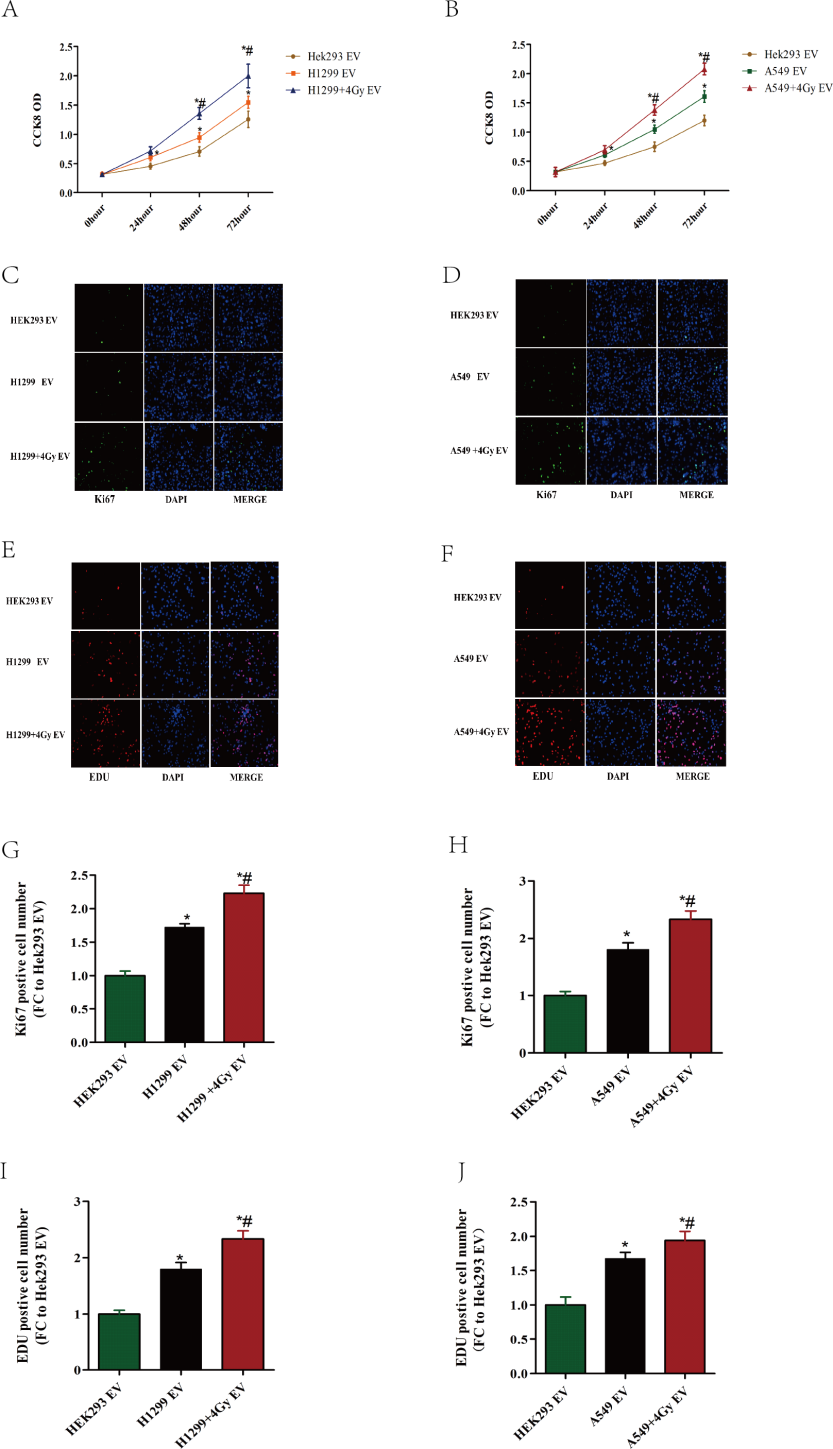
Lung cancer cell-derived EV enhance the proliferation of HUVECs. (A–J) HUVECs were incubated with EVs derived from different cells for the indicated times. The cells are HEK293, A549 and A549 exposed to 4 Gy X-ray irradiation, and H1299 and H1299 exposed to 4 Gy X-ray irradiation. (A, B) The CCK8 assay was performed to evaluate the effect of EVs on endothelial cell proliferation. (C, D) The proliferating HUVECs were labeled with EdU. The click-it reaction revealed the EdU staining (red). The cell nuclei were stained with DAPI (blue). The images are the representative of the results. (E, F) Immunofluorescence for Ki67 of the cells treated with the indicated EVs, Ki67 which represents the proliferative cells is shown in green, and the nuclei of endothelial cells are stained with DAPI (blue). The images are the representative of the results. (G, H) Quantification of the data from (C, D), which are expressed as the percentage of cells stained with EdU. The data are expressed as the fold change over the treatment of HEK293-derived EVs. (I, J) Quantification of the data from (G, H), which are expressed as the percentage of cells stained with Ki-67. The data are expressed as the fold change over the treatment of HEK293-derived EVs. ∗, *P* < 0.05, compared with the treatment of HEK293-derived EV. ∗#: *P* < 0.05, compared with the treatment of EVs derived from A549 or H1299 without exposure to irradiation.

A CCK-8 assay, EdU staining and Ki-67 immunofluorescence were performed to investigate the effect of EVs derived from different cell lines on the proliferation of HUVEC cells. We set HEK293 cell, a well-known cell biology research model, as control cell. Because we did not detect any difference in proliferation in HUVEC cells between HEK293 cell derived EVs and medium treated (Data S1). As shown in Fig. 2, compared with HEK293-derived EVs, EVs secreted by lung cancer cells (A549 or H1299) promoted the proliferation of HUVECs, and the effect was enhanced when the cancer cells were exposed to 4 Gy X-ray irradiation. These results demonstrated that EVs derived from lung cancer cells enhanced the proliferation of HUVECs, and this effect was strengthened when the cells were exposed to X-ray irradiation.

### The EVs isolated from irradiation-exposed lung cancer cells enhance the migration of endothelial cells

**Figure 3:**
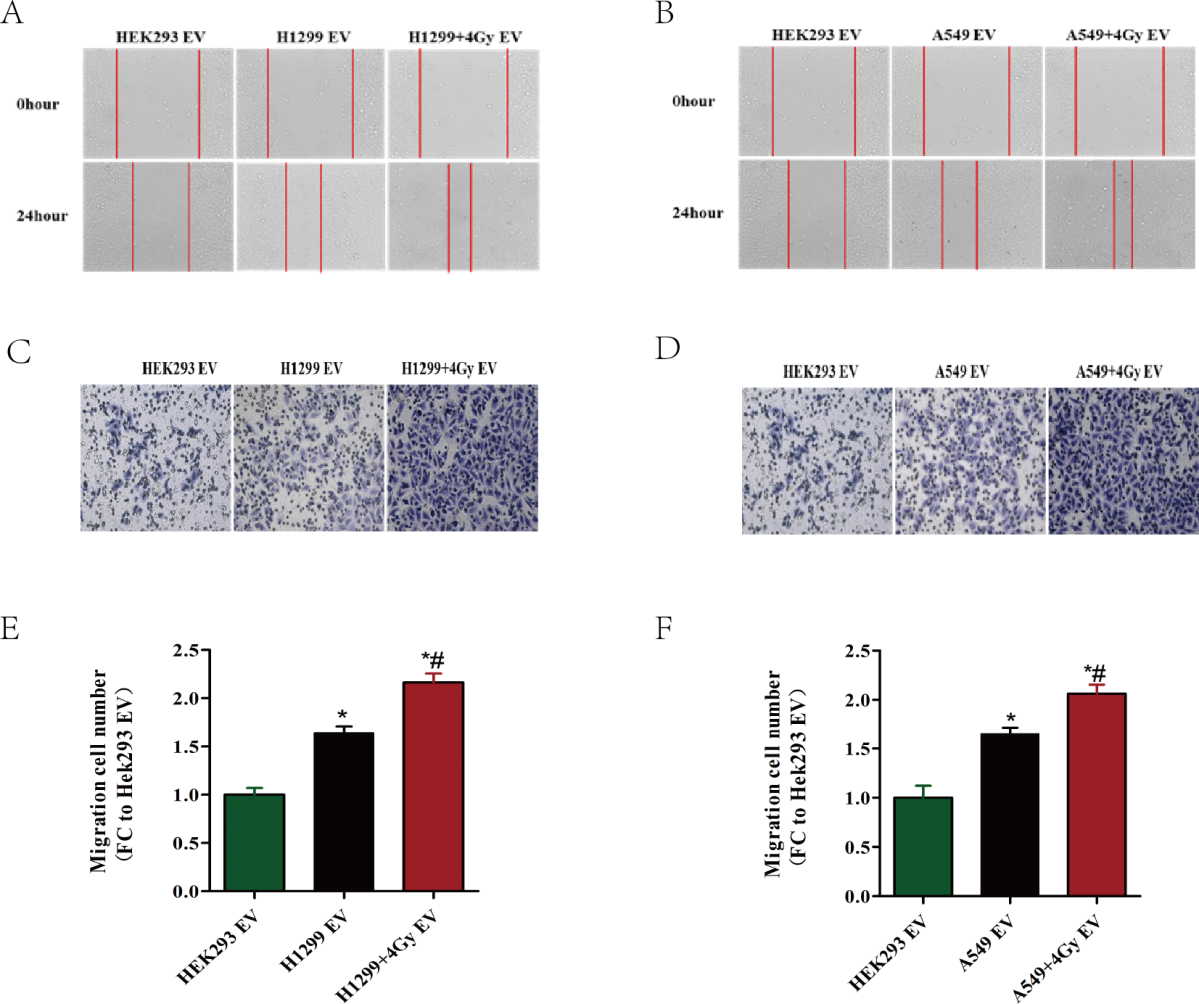
Lung cancer cell-derived EV enhance the migration of HUVECs. (A–F) HUVECs were incubated with EVs derived from different cells for the indicated time. The cells are HEK293, A549 or A549 exposed to 4 Gy X-ray irradiation, H1299 or H1299 exposed to 4 Gy X-ray irradiation. (A, B) Wound healing assay for HUVECs incubated with different sources of EVs. (C, D) Transwell assay for HUVECs incubated with different sources of EVs. The images are the representative of the results. (E, F) Quantification of the data from (C, D). ^∗^, *P* < 0.05, compared with the treatment of HEK293-derived EVs. ^∗^#:*P* < 0.05, compared with the treatment of EVs derived from A549 or H1299 without exposure to irradiation.

Wound healing assays and transwell assays were conducted to examine the migration of HUVECs exposed to different EVs. As shown in Fig. 3, EVs derived from lung cancer cells (A549 or H1299) remarkably accelerated the migration of HUVECs compared with EVs derived from HEK293 cells, which have been defined as a type of control cell. The migration capacity was further enhanced when A549 or H1299 cells were exposed to 4 Gy X-ray irradiation. These results indicated that EVs secreted by lung cancer cells could play an active role in radiation-induced endothelial cell migration.

### The downregulation of PTEN plays a key role in EVs-induced endothelial cells proliferation and migration

**Figure 4:**
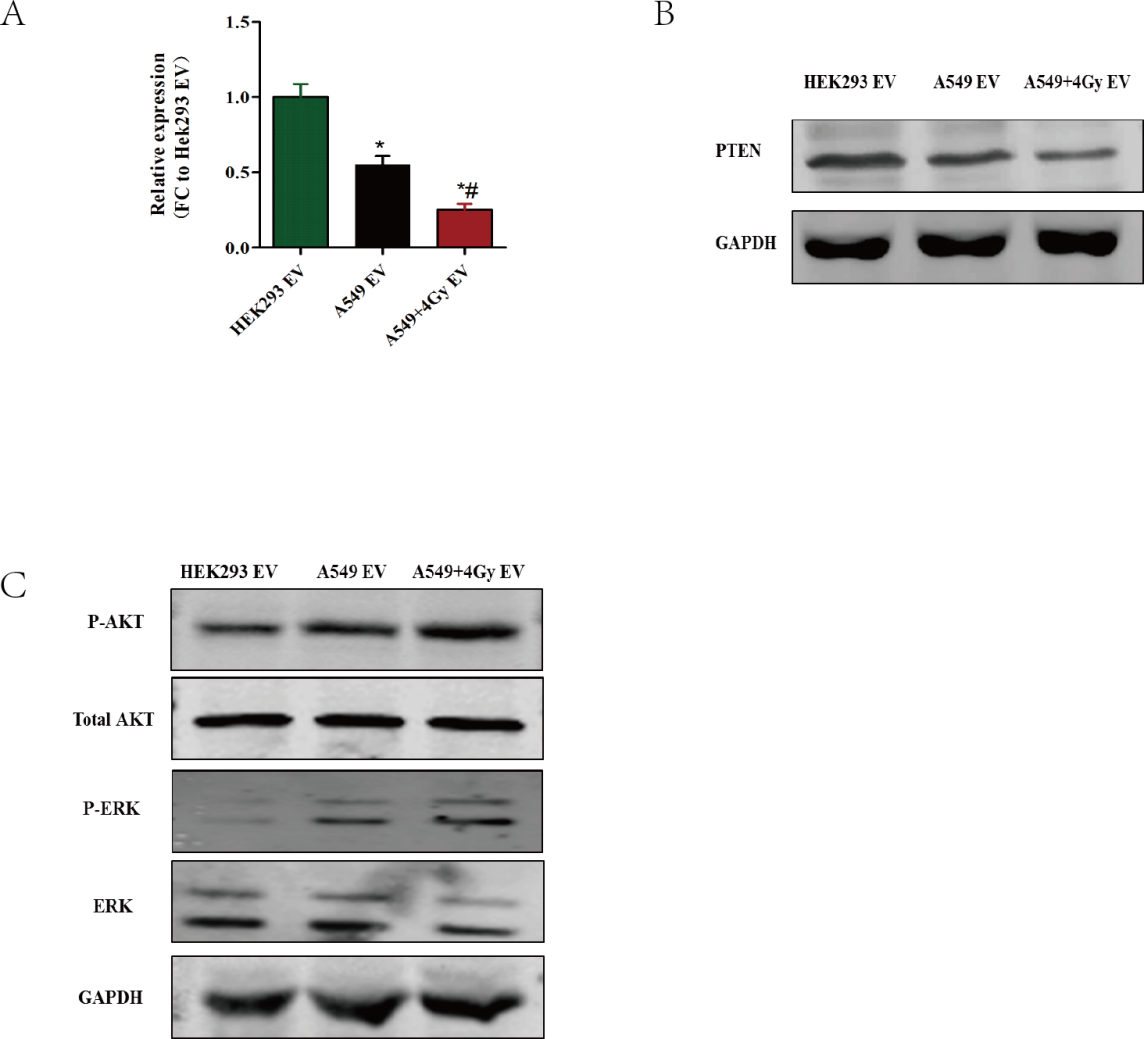
The downregulation of PTEN plays a key role in EV-induced endothelial cell proliferation and migration. HUVECs were incubated with EVs derived from different cells for the indicated times. The cells are HEK293, A549 and A549 exposed to 4 Gy X-ray irradiation. (A) After the cells were treated with the indicated EVs, the mRNA levels of PTEN were evaluated using qRT-PCR. The data are expressed as the fold change over the HUVECs treated with HEK293-derived EVs. ^∗^, *P* < 0.05, compared with the treatment of HEK293-derived EV. ^∗^#:*P* < 0.05, compared with the treatment of EVs derived from A549 or H1299 without exposure to irradiation. (B, C) HUVECs were treated with the indicated EVs. Then, the protein levels of PTEN (B) or p-AKT and p-ERK(C) were detected using western blotting, using GAPDH as a loading control.

The above results have suggested that lung cancer cell-derived EVs can promote the proliferation of HUVECs. For underlying the mechanisms that mediated this phenomenon, we tested many signaling molecules regulating proliferation and migration in A549 derived EVs treated HUVECs, including p53, p21, NF-κB, Hipo-YAP, JAK-stat, WNT and PTEN, and found that PTEN changed most significantly among them (Fig. 4B and Data S1). We also tested protein expression of these genes in H1299 derived EVs treated HUVECs, and found that trend is consistent with the A549 derived EVs treated HUVECs. Therefore, we selected A549 cell as model for further mechanism research. PTEN, a well-defined tumor suppressor gene, has been demonstrated to participate in cell cycle arrest. It has also been reported that deficiency of PTEN contributes to tumor cell migration and invasion. Our results in Fig. 4 show that compared with HEK293-derived EVs, A549-derived EVs remarkably decreased the level of PTEN in HUVECs and irradiation-exposed A549-derived EVs enhanced this effect (Figs. 4A and 4B), however PTEN expression (protein and mRNA) in A549 and A549-derived EVs remain unchanged (Data S1). To determine whether PTEN induced the function change in EVs-treated HUVECs, we detected the downstream target of PTEN, which is well known as AKT. As shown in Fig. 4C, A549-derived EVs remarkably increased the level of p-AKT and p-ERK, the active form of AKT and ERK, in HUVECs, with no significant effect in total AKT and ERK. These results indicate that the downregulation of PTEN plays a vital role in EVs-induced proliferation and migration.

### Transferred miR-23a mediates an EVs-induced reduction of PTEN in HUVECs

**Figure 5:**
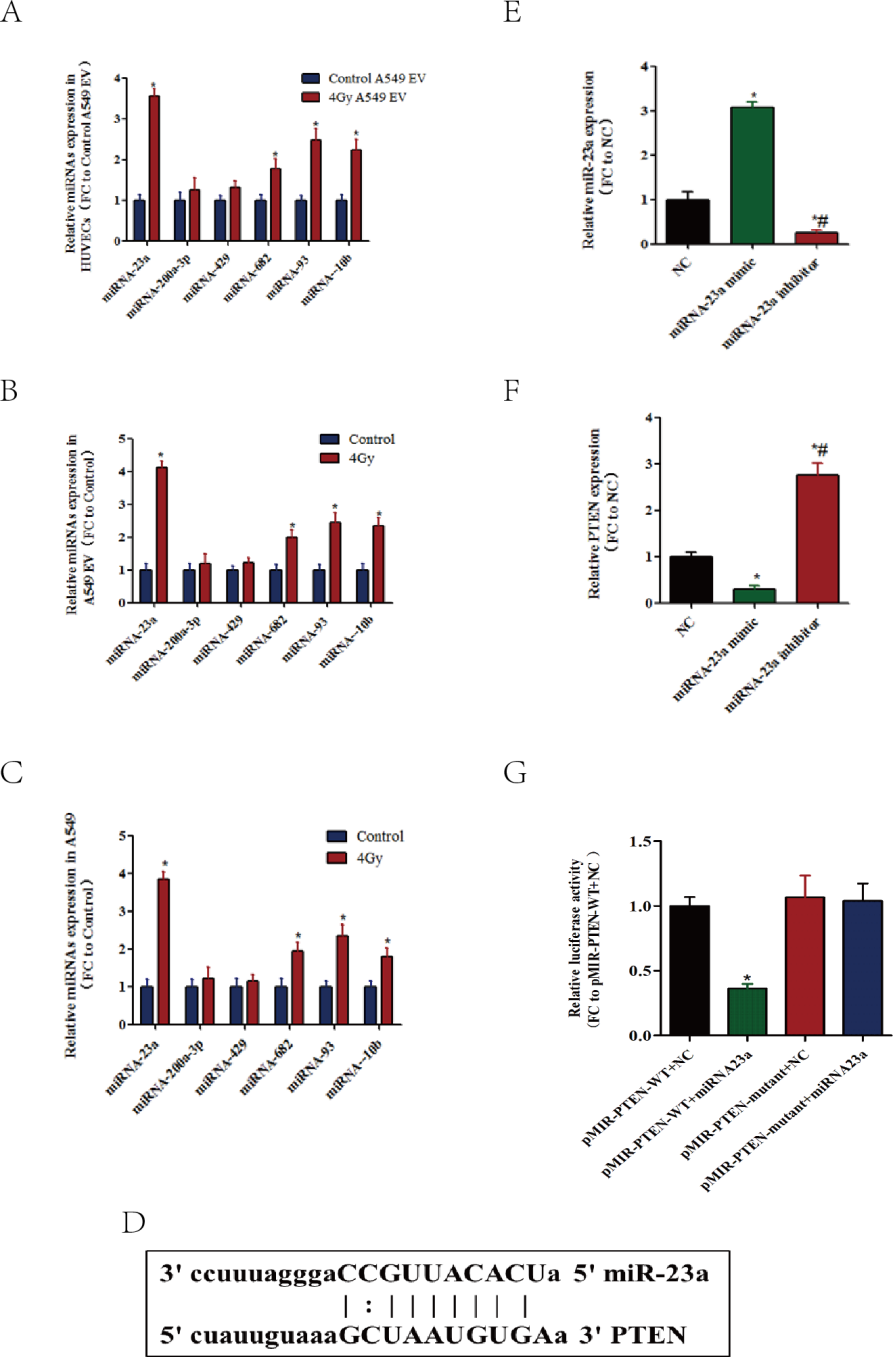
Transferred miR-23a mediates EV-induced downregulation of PTEN in HUVECs. (A) After being treated with the indicated EVs, the mRNA levels of HUVECs corresponding to miRNAs that could target PTEN were evaluated using qRT-PCR. The data are expressed as the fold change over the HUVECs treated with EVs from A549 cells without X-ray irradiation. (B) After being exposed to 4 X-ray irradiation, the EVs from A549 cells were isolated, and the levels of miRNAs that could target PTEN were evaluated by qRT-PCR. (C) After being exposed to 4 X-ray irradiation, the levels of miRNAs that could target PTEN were evaluated in A549 cells using qRT-PCR. (D) Schematic representation of a predicted binding site of miR-23a in the 3′-UTR of human PTEN mRNA. (E) Fold change of natural miR-23a when A549 cells were transfected with miR-23a mimics or inhibitor, detected with qRT-PCR. (F) Fold change of PTEN mRNA when A549 cells were transfected with miR-23a mimics or inhibitor, detected with qRT-PCR. (G) Luciferase reporter assay was performed in HEK293 cells by cotransfection of wild type PTEN-WT-3′-UTR vector or PTEN-mutant-3′-UTR vector and miR-23a mimics or negative control for 24 h. Then, the luciferase activity was determined using the Dual-Luciferase Reporter System, miR-23a mimics significantly suppressed the luciferase activity of the wild type reporter but minimal effect on the mutant reporter. ^∗^, *P* < 0.05.

It has been reported that EVs include various RNAs, particularly miRNAs. Previous studies demonstrated that EVs could accelerate the maturation of miRNAs, which can regulate target gene expression by mediating mRNA degradation or translational inhibition via specific binding to the 3′-UTR of mRNA. Therefore, we focused on EVs-transferred miRNAs involved in decreasing the level of PTEN. Predicted with three websites: miRanda (http://www.microrna.org/microrna/home.do), TargetScan (http://www.targetscan.org/vert_71/) and picTar (http://pictar.mdc-berlin.de/), we selected 6 miRNAs which were most probable miRNAs of the target gene PTEN according to the prediction score, and then tested their expression changes. Of the 6 miRNAs, miR-23a, miR-682, miR-93, and miR-10b level were markedly increased by 4 Gy X-ray irradiation-treated A549-derived EVs in HUVECs (Fig. 5A). When A549 cells were exposed to X-ray irradiation, miR-23a had the maximum increase in the A549 and A549 secreted EVs (Figs. 5B and 5C), indicating that the upregulated miR-23a suppresses the expression of PTEN.

Furthermore, bioinformatic analysis was done to predict the binding site of miR-23a in the 3′-UTR of PTEN mRNA (Fig. 5D). To determine whether miR-23a could affect the expression of PTEN, we over-expressed miR-23a through mimics in HEK293 cells. Indeed, as shown in Fig. 5E, transient transfection of the cells with miR-23a-specific mimics or inhibitors increased or decreased the level of miR-23a, respectively. Increased miR-23a with miR-23a mimics could downregulate the mRNA level of PTEN; meanwhile, decreased miR-23a had the opposite effect (Fig. 5F). A luciferase reporter assay showed that the miR-23a mimics caused a 65% reduction of luciferase activity of pmir-PTEN-wild type but minimal effect on the mutant reporter (Fig. 5G).

### Transferred miR-23a mediates EVs-induced HUVEC proliferation and migration

**Figure 6:**
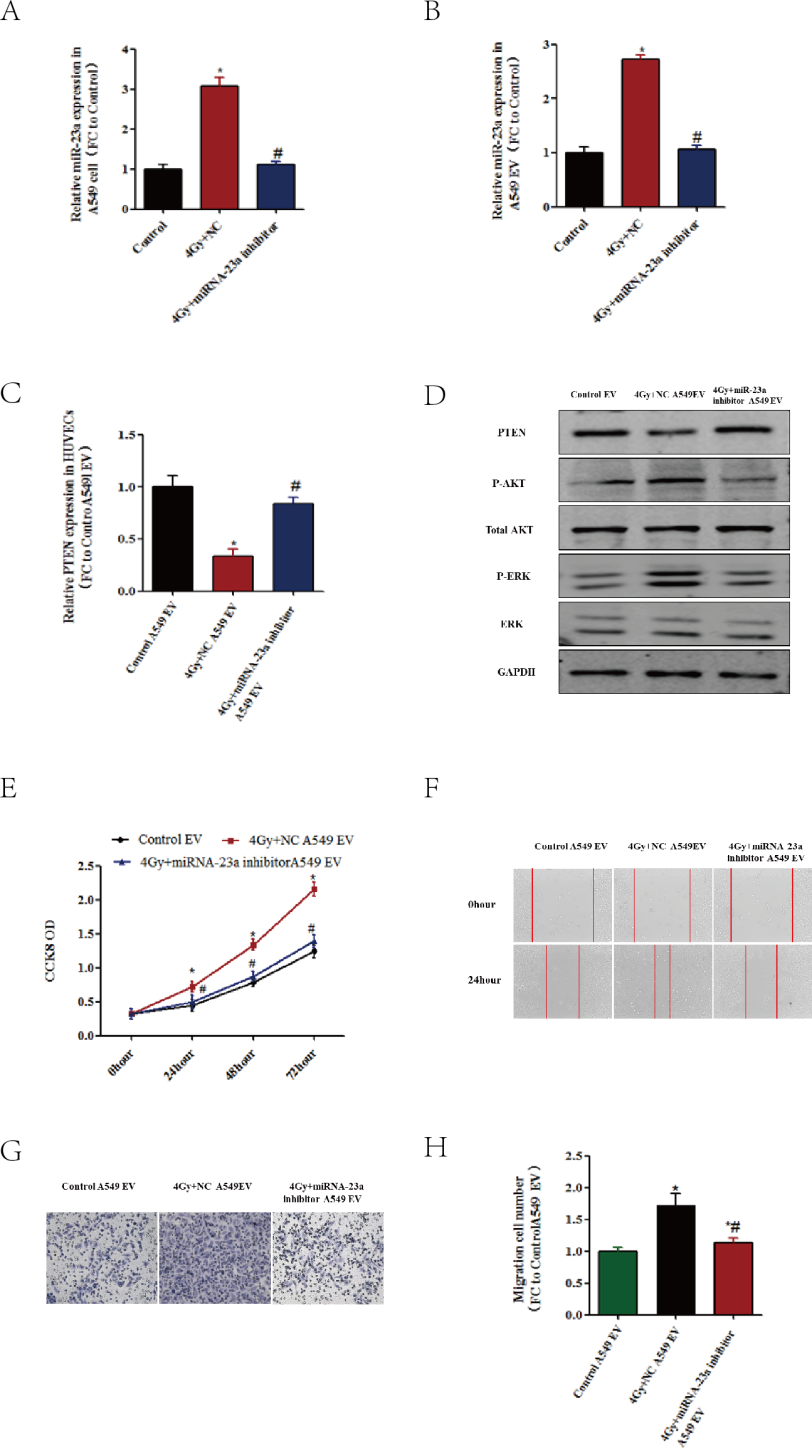
Transferred miR-23a mediates EV-induced HUVEC proliferation and migration. (A–B) After being exposed to 4 X-ray irradiation, with and without miR-23a inhibitor, the levels of natural miR-23a in A549cells were evaluated using qRT-PCR. (B) After being exposed to 4 X-ray irradiation, with and without miR-23a inhibitor, the EVs from A549cells were isolated, and the levels of miR-23a were evaluated using qRT-PCR. ^∗^, *P* < 0.05. compared with control. ^∗^#:*P* < 0.05, compared with 4 Gy. (C–H) After being exposed to 4 X-ray irradiation, with and without miR-23a inhibitor, the EVs were isolated and incubated with HUVECs. (C) The levels of PTEN mRNA in HUVECs were detected with qRT-PCR. (D) The protein levels of PTEN p-AKT and p-ERK were detected using western blotting, using GAPDH as a loading control. (E) The CCK8 assay was performed to evaluate the effect of EVs on endothelial cell proliferation. (F) Wound healing assay for HUVECs incubated with different sources of EVs. (G) Transwell assay for HUVECs incubated with different sources of EVs. The images are the representative of the results. (H) Quantification of the data from (G). ^∗^, *P* < 0.05. compared with control EVs. ^∗^#:*P* < 0.05, compared with 4 Gy +NC EVs.

To clarify whether miR-23a transferred by EVs affects HUVEC proliferation and migration, first, the variation of miR-23a in A549 was detected. As shown in Fig. 6A, X-ray irradiation markedly increased the miR-23a level, and an miR-23a inhibitor suppressed this phenomenon. The same effects were detected in A549-derived EVs (Fig. 6B). Incubated with A549-derived EVs, the mRNA level of PTEN in HUVECs had an inverse pattern to that of miR-23a levels (Fig. 6C). The protein level of PTEN and its activity (which is reflected in p-AKT) showed the same change (Fig. 6D).

Subsequently, we examined whether increases in miR-23a could increase the proliferation and migration of HUVECs. As shown in Figs. 6E–6G, the EVs derived from 4Gy X-ray-exposed A549 cells remarkably increased the proliferation and migration of HUVECs, whereas downregulation of miR-23a levels with its inhibitor reversed the radiation-induced proliferation and migration.

These results demonstrate that the miR-23a/PTEN pathway in EVs secreted by radiation induced lung cancer cells plays an important role in promoting endothelial cell angiogenesis.

## Discussion

To our knowledge, this is first to reveal that EVs-transferred miR-23a has the ability to accelerate the angiogenesis mediated by radiotherapy through inhibiting the expression of PTEN in HUVECs.

It has been demonstrated that angiogenesis facilitates tumor metastasis, and contributes to radiation resistance (Verma & Lautenschlaeger, 2016; Ochiai et al., 2016; Folkman, 1995; Li et al., 2016b). Among the factors that can affect vascular formation, small membrane-enclosed vesicles secreted by cancer cells have been demonstrated to be endocytosed by HUVECs and enhance the proliferation and migration of these cells. These small vesicles are called EVs.

Previous studies have reported that the contents of EVs are heterogeneous, including diverse RNA species, various proteins, including cytokines and growth factors, and lipids (Costa-Silva et al., 2015; Mulcahy, Pink & Carter, 2014). A number of miRNAs have been demonstrated to be present in EVs from cancer cells and serve essential roles in the regulation of tumor cell proliferation and migration (Urbich, Kuehbacher & Dimmeler, 2008). Among the miRNAs that can affect tumor metastasis, miR-23a has been shown to function as an oncogene (Lin et al., 2013; Chen et al., 2008). It is increased in various tumor tissues, such as adenocarcinoma, colorectal cancer and lung cancer (Kutay et al., 2006; Kulshreshtha et al., 2007). The expression level of miR-23a is also correlated with tumor metastasis and chemoradiation resistance (Cai et al., 2015). Results indicate that miR-23a may play an important role in EVs-mediated HUVEC proliferation and migration. In this study, we found that miR-23a was upregulated in HUVECs when the cells endocytosed EVs. Meanwhile, the increased miR-23a facilitates HUVEC proliferation and migration, which are necessary for angiogenesis.

MiRNAs are highly conserved noncoding RNAs (18∼24 nucleotides) that regulate target gene expression by mediating mRNA degradation or translational inhibition via specific binding to the 3′-UTR of mRNA (Zhou et al., 2014; Zhong et al., 2013). MiRNAs can act as tumor enhancers or suppressors and regulate up to 60% of protein-coding genes. In the present study, bioinformatics analysis predicts that miR-23a may target the PTEN 3′-UTR. It is well known that PTEN takes part in the PI3K/AKT pathway (Maehama & Dixon, 1998), which restrains cell proliferation and suppresses vascular formation (Chen et al., 2016); therefore, PTEN has been defined as a tumor suppressor. In our research, we demonstrated that miR-23a could downregulate PTEN expression in lung cancer cells by directly targeting the 3′-UTR of PTEN mRNA. The reduction in PTEN by miR-23a promoted HUVEC proliferation and migration.

In conclusion, the present study demonstrated that EVs secreted by lung cancer cells can promote angiogenesis. The secreted EVs transferred miR-23a into HUVECs, decreasing the level of the tumor suppressor PTEN and increasing cellular proliferation and migration. This pathway facilitates angiogenesis and radiation resistance, and it may be a novel target for the treatment of lung cancer.

##  Supplemental Information

10.7717/peerj.3627/supp-1 Data S1 Supplemental data Figure S1: Effect of HEK293,A549,H1299 cell derived EVs and medium on HUVECs proliferation. ^∗^, *P* < 0.05, compared with the treatment of HEK293-derived EV. ^∗^#:*P* < 0.05, compared with the treatment of EVs derived from A549 without exposure to irradiation.
Figure S2–S7: Western blot for genes sorting,S2:JAK expression; S3:p21 expression; S4:p53 expression; S5:p65 expression; S6:YAP expression; S7: *β*-catenin expression.S8: PTEN protein expression in A549 cell and EV;S9: PTEN mRNA expression in A549 cell and EV.

10.7717/peerj.3627/supp-2 Data S2 Raw data Raw data of the figures in the manuscript and relative original Western Blot.

## Additional Information and Declarations

### Data Availability

The following information was supplied regarding data availability: The raw data has been supplied as a Supplemental File.
