# Supplementary figures and images for: The extracellular vesicles secreted by lung cancer cells in radiation therapy promote endothelial cell angiogenesis by transferring miR-23a
Source: PeerJ. 2017 Aug 25;5:e3627. doi: 10.7717/peerj.3627 (PMC5572936; doi:10.7717/peerj.3627)

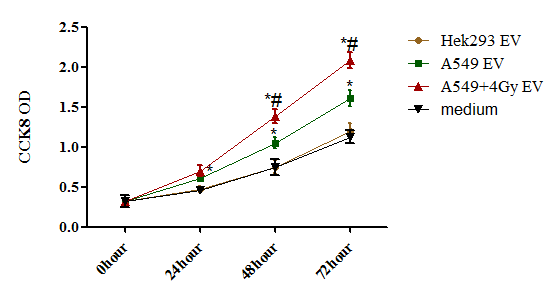

Supplement: Data S1 — Figure S1: Effect of HEK293,A549,H1299 cell derived EVs and medium on HUVECs proliferation. ∗, P < 0.05, compared with the treatment of HEK293-derived EV. ∗#:P < 0.05, compared with the treatment of EVs derived from A549 without exposure to irradiation. Figure S2–S7: Western blot for genes sorting,S2:JAK expression; S3:p21 expression; S4:p53 expression; S5:p65 expression; S6:YAP expression; S7: β-catenin expression.S8: PTEN protein expression in A549 cell and EV;S9: PTEN mRNA expression in A549 cell and EV. [file peerj-05-3627-s001.zip › supplement data/S1ú║effect of HEK293,A549,H1299 cell derived EVs and medium on HUVECs proliferation.tif]

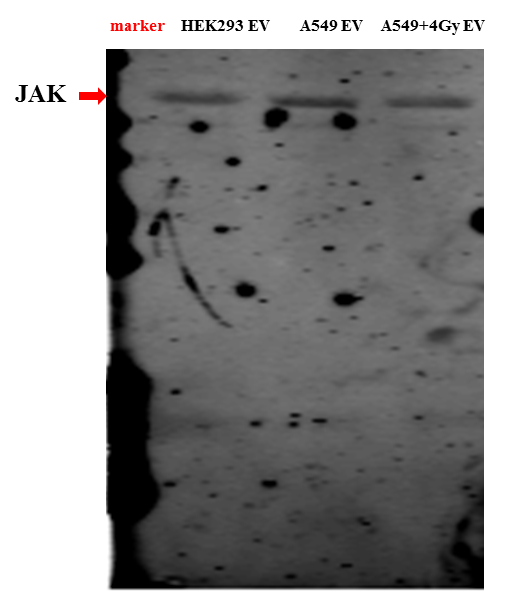

Supplement: Data S1 — Figure S1: Effect of HEK293,A549,H1299 cell derived EVs and medium on HUVECs proliferation. ∗, P < 0.05, compared with the treatment of HEK293-derived EV. ∗#:P < 0.05, compared with the treatment of EVs derived from A549 without exposure to irradiation. Figure S2–S7: Western blot for genes sorting,S2:JAK expression; S3:p21 expression; S4:p53 expression; S5:p65 expression; S6:YAP expression; S7: β-catenin expression.S8: PTEN protein expression in A549 cell and EV;S9: PTEN mRNA expression in A549 cell and EV. [file peerj-05-3627-s001.zip › supplement data/S2ú║JAK.tif]

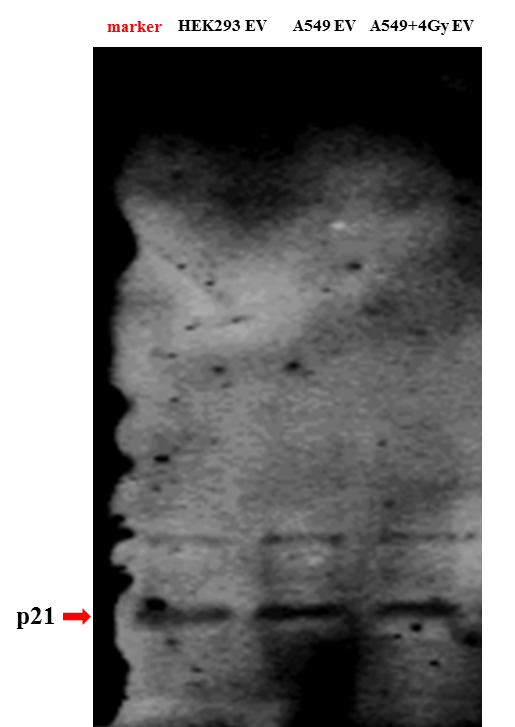

Supplement: Data S1 — Figure S1: Effect of HEK293,A549,H1299 cell derived EVs and medium on HUVECs proliferation. ∗, P < 0.05, compared with the treatment of HEK293-derived EV. ∗#:P < 0.05, compared with the treatment of EVs derived from A549 without exposure to irradiation. Figure S2–S7: Western blot for genes sorting,S2:JAK expression; S3:p21 expression; S4:p53 expression; S5:p65 expression; S6:YAP expression; S7: β-catenin expression.S8: PTEN protein expression in A549 cell and EV;S9: PTEN mRNA expression in A549 cell and EV. [file peerj-05-3627-s001.zip › supplement data/S3ú║p21.tif]

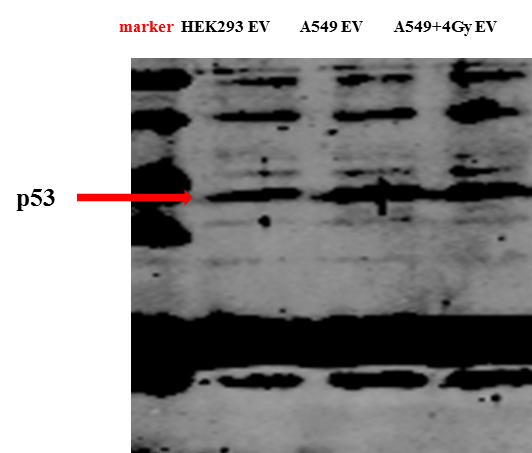

Supplement: Data S1 — Figure S1: Effect of HEK293,A549,H1299 cell derived EVs and medium on HUVECs proliferation. ∗, P < 0.05, compared with the treatment of HEK293-derived EV. ∗#:P < 0.05, compared with the treatment of EVs derived from A549 without exposure to irradiation. Figure S2–S7: Western blot for genes sorting,S2:JAK expression; S3:p21 expression; S4:p53 expression; S5:p65 expression; S6:YAP expression; S7: β-catenin expression.S8: PTEN protein expression in A549 cell and EV;S9: PTEN mRNA expression in A549 cell and EV. [file peerj-05-3627-s001.zip › supplement data/S4ú║p53.tif]

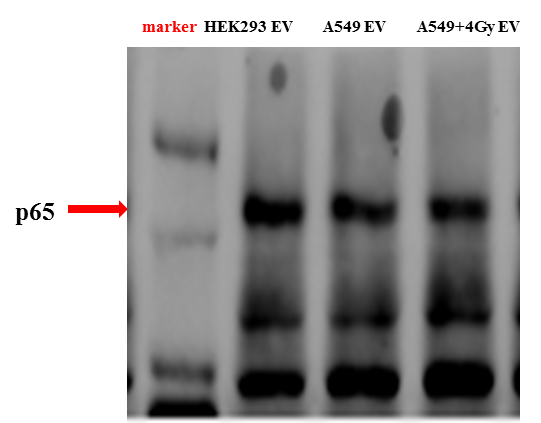

Supplement: Data S1 — Figure S1: Effect of HEK293,A549,H1299 cell derived EVs and medium on HUVECs proliferation. ∗, P < 0.05, compared with the treatment of HEK293-derived EV. ∗#:P < 0.05, compared with the treatment of EVs derived from A549 without exposure to irradiation. Figure S2–S7: Western blot for genes sorting,S2:JAK expression; S3:p21 expression; S4:p53 expression; S5:p65 expression; S6:YAP expression; S7: β-catenin expression.S8: PTEN protein expression in A549 cell and EV;S9: PTEN mRNA expression in A549 cell and EV. [file peerj-05-3627-s001.zip › supplement data/S5ú║p65.tif]

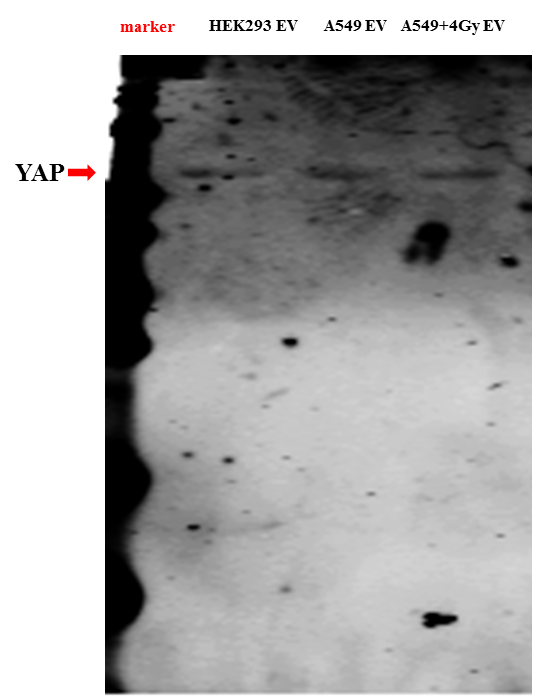

Supplement: Data S1 — Figure S1: Effect of HEK293,A549,H1299 cell derived EVs and medium on HUVECs proliferation. ∗, P < 0.05, compared with the treatment of HEK293-derived EV. ∗#:P < 0.05, compared with the treatment of EVs derived from A549 without exposure to irradiation. Figure S2–S7: Western blot for genes sorting,S2:JAK expression; S3:p21 expression; S4:p53 expression; S5:p65 expression; S6:YAP expression; S7: β-catenin expression.S8: PTEN protein expression in A549 cell and EV;S9: PTEN mRNA expression in A549 cell and EV. [file peerj-05-3627-s001.zip › supplement data/S6ú║YAP.tif]

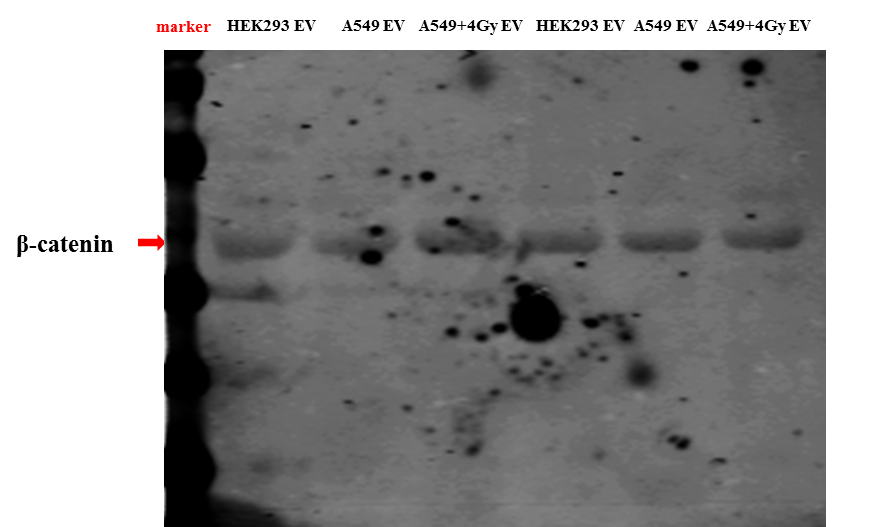

Supplement: Data S1 — Figure S1: Effect of HEK293,A549,H1299 cell derived EVs and medium on HUVECs proliferation. ∗, P < 0.05, compared with the treatment of HEK293-derived EV. ∗#:P < 0.05, compared with the treatment of EVs derived from A549 without exposure to irradiation. Figure S2–S7: Western blot for genes sorting,S2:JAK expression; S3:p21 expression; S4:p53 expression; S5:p65 expression; S6:YAP expression; S7: β-catenin expression.S8: PTEN protein expression in A549 cell and EV;S9: PTEN mRNA expression in A549 cell and EV. [file peerj-05-3627-s001.zip › supplement data/S7ú║a┬-catenin.tif]

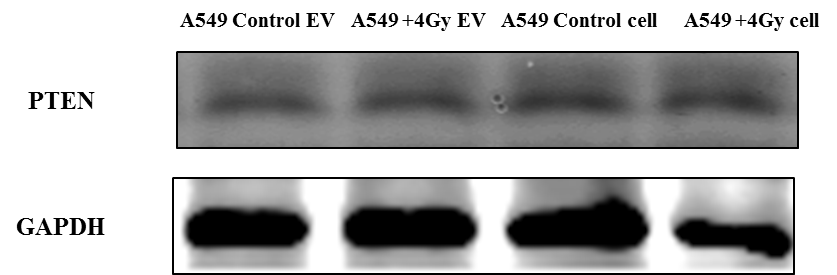

Supplement: Data S1 — Figure S1: Effect of HEK293,A549,H1299 cell derived EVs and medium on HUVECs proliferation. ∗, P < 0.05, compared with the treatment of HEK293-derived EV. ∗#:P < 0.05, compared with the treatment of EVs derived from A549 without exposure to irradiation. Figure S2–S7: Western blot for genes sorting,S2:JAK expression; S3:p21 expression; S4:p53 expression; S5:p65 expression; S6:YAP expression; S7: β-catenin expression.S8: PTEN protein expression in A549 cell and EV;S9: PTEN mRNA expression in A549 cell and EV. [file peerj-05-3627-s001.zip › supplement data/S8ú║PTEN protein expression in A549 cell and EV.tif]

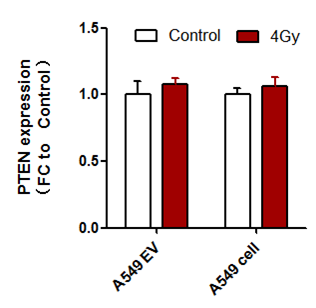

Supplement: Data S1 — Figure S1: Effect of HEK293,A549,H1299 cell derived EVs and medium on HUVECs proliferation. ∗, P < 0.05, compared with the treatment of HEK293-derived EV. ∗#:P < 0.05, compared with the treatment of EVs derived from A549 without exposure to irradiation. Figure S2–S7: Western blot for genes sorting,S2:JAK expression; S3:p21 expression; S4:p53 expression; S5:p65 expression; S6:YAP expression; S7: β-catenin expression.S8: PTEN protein expression in A549 cell and EV;S9: PTEN mRNA expression in A549 cell and EV. [file peerj-05-3627-s001.zip › supplement data/S9ú║PTEN mRNA expression in A549 cell and EV.tif]
